# Co‐Occurrence of *SCN9A* and *PRRT2* Variants in a Patient With Paroxysmal Extreme Pain Disorder, Contradictory Analgesia, and Intractable Paroxysmal Non‐Kinesigenic Dyskinesia

**DOI:** 10.1155/carm/5942985

**Published:** 2026-06-22

**Authors:** Miki Ikeda, Aritomo Kawashima, Kaori Kodama, Ryo Sato, Saki Uneoka, Yu Katata, Yukimune Okubo, Wakaba Endo, Takehiko Inui, Noriko Togashi, Chika Mizuno, Hisao Yaoita, Naoya Saijo, Jun Takayama, Gen Tamiya, Atsuo Kikuchi, Shigeo Kure, Kazuhiro Haginoya

**Affiliations:** ^1^ Department of Pediatric Neurology, Miyagi Children’s Hospital, Sendai, 989-3126, Japan, miyagi-children.or.jp; ^2^ Department of Orthopedic Surgery, Miyagi Children’s Hospital, Sendai, 989-3126, Japan, miyagi-children.or.jp; ^3^ Department of Pediatrics, Tohoku University School of Medicine, Sendai, 980-8574, Japan, tohoku.ac.jp; ^4^ Department of AI and Innovative Medicine, Tohoku University Graduate School of Medicine, Sendai, 980-8575, Japan, tohoku.ac.jp; ^5^ Tohoku Medical Megabank Organization, Tohoku University, Sendai, 980-8573, Japan, tohoku.ac.jp; ^6^ Statistical Genetics Team, RIKEN Center for Advanced Intelligence Project, Tokyo, 103-0027, Japan, riken.jp; ^7^ Department of Rare Disease Genomics, Tohoku University Graduate School of Medicine, Sendai, 980-8573, Japan, tohoku.ac.jp

**Keywords:** channelopathy-associated insensitivity to pain, I234T variant, paroxysmal extreme pain disorder, paroxysmal non-kinesigenic dyskinesia, primary erythromelalgia, *PRRT2*, *SCN9A*

## Abstract

Gain‐of‐function mutations in *SCN9A*, encoding the voltage‐dependent Nav1.7 sodium channel, cause three autosomal‐dominant disorders associated with severe pain: primary erythromelalgia, paroxysmal extreme pain disorder (PEPD), and small fiber neuropathy. On the other hand, biallelic loss‐of‐function mutations have been linked to impaired pain perception. Notably, the coexistence of both hyperalgesia and hypoalgesia within the same patient harboring the I234T variant has been reported in three independent patients to date. We report a 7‐year‐old girl harboring co‐occurring *SCN9A* (I234T) and *PRRT2* variants who presented with paroxysmal extreme pain disorder, contradictory analgesia, sensitivity to heat, and intractable head‐drop attacks. Based on the genetic and clinical analyses, she was diagnosed as having PEPD and *PRRT2*‐related paroxysmal dyskinesia. The intractable head‐drop attacks were considered as paroxysmal non‐kinesigenic dyskinesia. In addition, she exhibited easy fatigability and hypotonia. Taken together with her cold, cyanotic feet, these findings suggest that she may have also had small fiber neuropathy.

## 1. Introduction

To date, nine types of voltage‐gated sodium channel α subunits are known, among which the Nav1.7 channel encoded by the *SCN9A* gene is preferentially expressed in the neurons of the sympathetic and dorsal root ganglia (DRG) [1]. Most DRG neurons serve as pain‐sensing (nociceptive) neurons and, thus, are involved in the pathophysiology of pain [1].

Gain‐of‐function mutations in *SCN9A* cause three autosomal‐dominant disorders associated with severe pain: primary erythromelalgia (PE; MIM#133020), paroxysmal extreme pain disorder (PEPD; MIM#167400; [2]), and small fiber neuropathy (SFN; [3]). PE is a rare disorder characterized by recurrent burning pain and redness in the extremities (predominantly the feet) triggered by warmth or exercise. It can present in childhood or adolescence but is more common in adults. PEPD is characterized by attacks of severe rectal, ocular, or submandibular pain accompanied by skin flushing [2, 4, 5]. Its onset usually occurs during the neonatal period or infancy. SFN is a relatively common disease of thin myelinated (Aδ fibers) and unmyelinated (C fibers) nerve fibers, clinically characterized by adult‐onset neuropathic pain and autonomic symptoms [3].

On the other hand, biallelic loss‐of‐function mutations have been linked to impaired pain perception associated with channelopathy‐associated insensitivity to pain (CIP; MIM#243000; [6]). CIP is a rare autosomal‐recessive disorder characterized by a complete lack of pain perception including noxious stimuli, but other sensations are preserved [6].

Among the *SCN9A* variants, p.I234T has been reported in three independent patients with unique characteristics [7–9], who presented with complex clinical phenotypes, including a clinical gain‐of‐function (similar to PE or PEPD) together with an impaired ability to sense pain, such as painless scratching, tooth extraction [8], self‐mutilation [7], and corneal anesthesia [9]. However, the neurological details of such patients have not been described.

*PRRT2* has been identified as a causative gene for benign familial infantile convulsions and paroxysmal kinesigenic dyskinesia [10]. We report a 7‐year‐old girl with a complex constellation of clinical manifestations, including PEPD and insensitivity to pain associated with the *SCN9A* (I234T) variant, and frequent head‐drop attacks likely attributable to the *PRRT2* variant.

## 2. Case Report

A girl aged 7 years and 4 months had been born via cesarean section after 36 weeks of pregnancy to nonconsanguineous Japanese parents. She lacked asphyxia. The birth weight, body length, and head circumference were 2218 g (−2.0 standard deviation [SD]), 47.0 cm (−0.7 SD), and 32.0 cm (−0.6 SD), respectively. Her mother had a history of convulsions in infancy. Her elder sister had been diagnosed with epilepsy at the age of 1 year and had been treated with an anticonvulsant for 2 years. Her maternal uncle had a history of epilepsy.

Her neonatal sucking ability was weak. Commencing at 2 months of age, she developed recurrent seizure‐like attacks; she suddenly cried and stiffened her limbs as if she had been startled. The attacks were particularly frequent when the body was warmed, such as during a bath. Her early development was delayed: head control at 6 months, rolling over at 10 months, and sitting alone at 10 months. At this time, sudden head‐drop attacks developed and she was referred to our hospital at 11 months of age.

**FIGURE 1:**
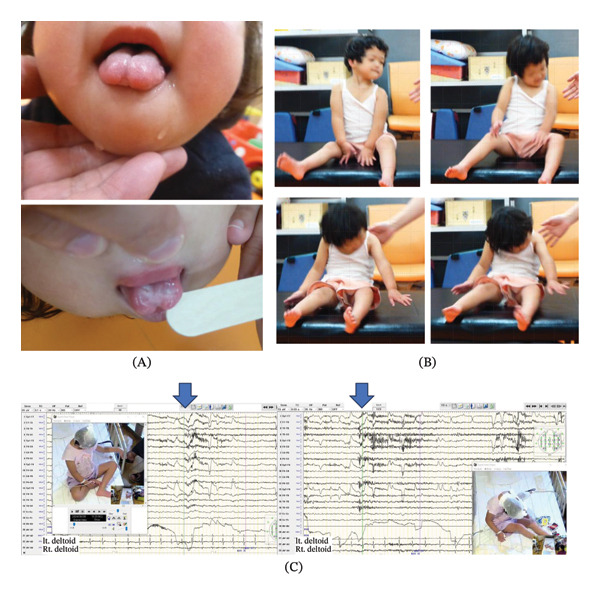
Tongue mutilation (A), head drop attack (B), and video‐EEG monitoring (C). A tongue deficit was first identified during a routine dental examination and was attributed to self‐inflicted tongue mutilation (A). Recurrent head‐drop episodes were observed clinically (B), some of which were followed by painful attacks. Video‐EEG monitoring at 7 years of age revealed frequent head‐drop attacks without discernible epileptiform discharges (C).

On examination, she exhibited a downslanted palpebral fissure. Neurologically, she presented with hypotonia, weak or absent deep tendon reflexes, but no pathological reflexes or spasticity. Sudden head‐dropping was observed on some occasions on the first visit but was not associated with sudden crying. She began to walk independently around 2 years of age, at which time she still exhibited hypotonia of the extremities and frequent sudden crying attacks. After she was able to talk, she started to complain of abrupt pain attacks in her anus followed by pain in her legs 4‐5 times a day. Her head‐drop attacks also continued at a rate of 10–20/day, in addition to the painful attacks. Notably, she was found to have a painless tongue bite at the age of 2 years (Figure 1A). A tibial bone fracture had occurred but was noticed later, at the age of 4 years. According to her mother, she did not cry during venipuncture, indicating that she was insensitive to pain. The patient also exhibited cyanotic and cold feet, suggesting peripheral circulatory insufficiency, from the age of 3 years and 6 months, but sweating appeared to be normal. Her IQ was 74 on the Tanaka–Binet test at 5 years and 10 months of age. Her short stature (HT, −2.8 SD and BW, −1.56 SD) was remarkable but the growth hormone secretion test was normal.

General biochemical blood tests and magnetic resonance imaging of the brain and spinal cord, performed at 2 years and 5 months of age, were unremarkable. Video electroencephalographic (EEG) monitoring performed at 2 years and 5 months of age detected a few sudden crying attacks with tonic posturing but no epileptic abnormalities. Neurophysiological examinations were conducted at 3 years and 1 month of age. The motor nerve conduction velocities and compound muscle action potentials were unremarkable for the median (MN), posterior tibial, and peroneal nerves. The sensory nerve conduction velocities were 52.5 m/s and 57.7 m/s for the median and sural nerve (SN), respectively. The sensory nerve action potentials were 32 μV and 9.9 μV for the MN and SN, respectively. The distal latency of the MN and SN were 1.62 and 2.66 ms, respectively. All sensory nerve parameters were considered unremarkable.

We performed family‐based whole‐exome sequencing at the age of 4 years and 5 months, as described previously [11]; we sought to establish a molecular diagnosis. The patient and her family provided informed consent for next‐generation sequencing and publication of this case report. The study was approved by the Ethics Review Boards of Miyagi Children’s Hospital and Tohoku University Hospital. We identified a *de novo* heterozygous variant in *SCN9A* c.701T > C (p.I234T) [NM_001365536.1], which was classified as “pathogenic” by the guidelines of the American College of Medical Genetics. In addition, a heterozygous pathogenic variant in *PRRT2* c.649dup (p.R217fs) [NM_145239.3]—a recognized hotspot for benign familial infantile convulsions and paroxysmal kinesigenic dyskinesia—was detected in the patient and her mother [10]. No other pathogenic variants were detected. Based on these results, she was diagnosed as having PEPD and *PRRT2*‐related paroxysmal dyskinesia.

Levetiracetam was not effective. Gabapentin alleviated the nighttime attacks and allowed her to sleep until morning but was discontinued because of increased daytime sleepiness (a side effect). Mexiletine was partially effective. After switching to carbamazepine (CBZ; 10 mg/kg/day), the painful attacks disappeared for 1 week; however, the attacks then reappeared. A subsequent trial of the sodium channel blocker lacosamide (45 mg/day; 2.6 mg/kg/day) produced no therapeutic benefit and, in fact, exacerbated the symptoms. Consequently, CBZ monotherapy was reinstated. At a dose of up to 12 mg/kg/day, corresponding to a serum concentration of 6.4 μg/mL, her painful attacks subsided; however, the head‐drop attacks persisted. Repeat video‐EEG monitoring after lacosamide treatment at age 7 years revealed frequent head‐drop attacks (50 episodes over 3.5 h) unassociated with epileptiform discharges but accompanied by truncal muscle contractions (Figure 1B). These attacks showed no diurnal fluctuation or age‐dependent evolution. A few brief painful attacks lasting 10–20 s were observed without overt epileptic activity, and some painful episodes occurred immediately after head‐drop attacks. Currently, she exhibits daily head‐drop attacks (10 times/day) (Figure 1C) without precipitating factors, as well as weak pain attacks involving the anus and lower extremities (particularly the ankles), areflexia, short stature, hypotonus of the extremities, cold and cyanotic feet, and an autistic tendency. Easy fatigability is also evident; a wheelchair is needed during long outings. She can climb only a few steps. She is sensitive to heat; air conditioning is essential for daily living.

## 3. Discussion

PE and PEPD are both associated with pain and flushing of the skin. However, various clinical symptoms and responses to medication have been reported. PEPD develops in the neonatal period and is essentially a visceral pain condition that is often unprovoked, with marked cranial nerve involvement. PE develops principally in adult life, primarily affects the extremities, and is sometimes triggered by temperature changes and exercise. CBZ is effective in many patients with PEPD but is ineffective or minimally effective in those with PE [12, 13].

Our patient’s symptoms were principally those of PEPD, but contradictory symptoms similar to analgesia (a tongue bite, a tibial fracture, and venipuncture without pain) were also observed. In previous reports of patients with the I234T variant, the first symptoms appeared at 11 months of age (breath‐holding spells) in one patient [7], at 3 months of age in another (unexplained bouts of irritability, excessive crying, and cyanotic spells during feeding) [8], and at 28 months of age in a third (daily pain attacks preceded by recurrent eye rubbing) [9]. All four patients, including our patient, reported warmth‐induced pain attacks similar to those seen in PE [7, 8]. However, our patient did not exhibit skin flushing during attacks.

Nonepileptic tonic seizures are among the most striking clinical symptoms in patients with PEPD [13–15]. Our patient first developed recurrent seizure‐like attacks at 2 months of age, making it difficult to determine whether these symptoms were attributable to the *PPRT2* variant or represented an early manifestation of PEPD. Furthermore, recent reports indicated the expanding clinical spectrum of *PRRT2*‐related phenotypes such as paroxysmal non‐kinesigenic dyskinesia and paroxysmal hypnogenic dyskinesia [16]. Her intractable head‐drop attacks were considered as paroxysmal non‐kinesigenic dyskinesia. Notably, some pain attacks occurred immediately after head‐drop episodes, suggesting that both phenomena may share a common pathophysiological mechanism. PRRT2 protein interacts with SNAP25, which has been implicated in neuropathic pain [17]. Interestingly, all three previously reported individuals harboring the I234T *SCN9A* variant exhibited erythromelalgia, a phenotype generally considered more severe than typical PEPD. It is conceivable that the *PRRT2* variant, or other genetic or epigenetic modifiers, may have influenced the *SCN9A*‐related phenotype in the present patient.

The patient had easy fatigability and heat sensitivity, which were persistently observed and were not related to her paroxysmal symptoms. Taken together with her cold, cyanotic feet, these findings suggest that she may have also had SFN [3, 18]. The clinical divergence between reduced nociceptive responses (hypoalgesia) and enhanced thermal sensitivity (hyperalgesia) may reflect altered Nav1.7 channel properties in response to noxious stimuli, potentially in a temperature‐dependent manner [19]. Furthermore, because self‐injurious behavior has also been observed in neuropathy [20], it remains unclear whether her symptoms were due to dysesthesia or discomfort secondary to peripheral neuropathies. The loss of deep tendon reflexes suggests neuromotor dysfunction, which requires further clarification.

Recent investigations have demonstrated that the contradictory clinical features associated with the I234T variant may reflect altered electrophysiological properties of the Nav1.7 channel, characterized by enhanced depolarization (hyperalgesia) followed by deeper or prolonged repolarization (hypoalgesia) [21]. In terms of treatments for patients with the I234T variant, one study reported that CBZ partly normalized the hyperpolarized activation of the mutant channel in I234T‐transfected DRG neurons [19]. Clinically, CBZ was effective in one case [8] but was withdrawn because of side effects in another [7]. The present patient exhibited a better therapeutic response to CBZ than to lacosamide or mexiletine, a finding that warrants further investigation.

## 4. Conclusion

We report a patient with co‐occurrence of *SCN9A* and *PRRT2* variants with paroxysmal extreme pain disorder and contradictory analgesia as well as intractable paroxysmal non‐kinesigenic dyskinesia. Some painful attacks occurred after head‐drop attacks, suggesting that the two phenomena may share a common pathophysiological mechanism. Further delineation of her symptoms is essential to optimize pharmacologic management and provide appropriate guidance for the family.

## Funding

Drs. Jun Takayama, Gen Tamiya, and Atsuo Kikuchi have received a research grant from Astellas Pharma.

## Consent

Written consent for presenting the photographs was obtained from the patient’s family.

## Conflicts of Interest

The authors declare no conflicts of interest.

## Data Availability

The data that support the findings of this study are available from the corresponding author upon reasonable request.
